# Targeted Gas Chromatography‐Mass Spectrometry Analysis of 31 Phthalates and Replacements: Method Optimization and Application to Edible Oils and Silicone Wristbands

**DOI:** 10.1002/jssc.70227

**Published:** 2025-07-17

**Authors:** Kaley T. Adams, Caoilinn Haggerty, Richard P. Scott, Steven O'Connell, Kim A. Anderson

**Affiliations:** ^1^ Department of Environmental and Molecular Toxicology Oregon State University Corvallis Oregon USA

**Keywords:** alternatives, gas chromatography, GC‐MS, mass spectrometry, phthalates, silicone wristbands, SPE

## Abstract

Interest in phthalate detection of foods and other environmental media has grown rapidly in the past decade. However, current analytical and separation techniques are often limited in the breadth of chemistry targeted, most often targeting less than 15 compounds. Challenges to successful methods with this compound group include chromatographic resolution, quantitation across diverse concentration ranges, and sample preparation due to the chemical similarity of these compounds. This project describes the development of a selective ion monitoring gas chromatography mass spectrometry method for quantitation of 29 phthalates and two phthalate replacements along with considerations for quantitation, sample cleanup, and standard storage. Our range of phthalates includes less‐studied ones like bis(2‐propylheptyl), diundecyl, didecyl, and ditridecyl. Analytical performance included limits of detection ranging from 17–230 ng/mL and robust reproducibility with relative percent differences below 8% for complex matrices. Two calibration ranges were used to accommodate the wide dynamic range of phthalate concentrations observed in real samples. Method application was demonstrated with edible oils (*n* = 12) and silicone wristbands (*n* = 18), representing dietary and personal exposure pathways. Sample preparation strategies, including solid phase extraction were evaluated to mitigate matrix interferences. In addition, compound storage stability was assessed over 133 days to inform best practices for standard preparation and handling. The finalized method demonstrates the uniquely large compound ranges for some phthalates and the importance of analyzing a wide variety of these compounds, making it a valuable foundation for comprehensive environmental monitoring of phthalates and their alternatives.

**Abbreviations**
BBP: butyl benzyl phthalate
CAS: chemical abstract service
DALP: diallyl phthalate
DAP: diamyl phthalate
DBEP: bis(2‐butoxyethyl) phthalate
DBzP: dibenzyl phthalate
DCHP: dicyclohexyl phthalate
DDP: didecyl phthalate
DEEP: bis(2‐ethoxyethyl) phthalate
DEHA: di(2‐ethylhexyl) adipate
DEHiP: bis(2‐ethylhexyl) isophthalate
DEHP: bis(2‐ethylhexyl) phthalate
DEHT: bis(2‐ethylhexyl) terephthalate
DEP: diethyl phthalate
DHtP: diheptyl phthalate
DiBP: di‐iso‐butyl phthalate
DiPP: diisopentyl phthalate
DiPrP: diisopropyl phthalate
DMEP: bis(2‐methoxyethyl) phthalate
DMP: dimethyl phthalate
DMPP: bis(4‐methylpentyl) phthalate
DnBP: di‐n‐butyl phthalate
DnHxP: di‐n‐hexyl phthalate
DnNP: di‐n‐nonyl phthalate
DnOP: di‐n‐octyl phthalate
DPhiP: diphenyl isophthalate
DPhP: diphenyl phthalate
DPrHtP: bis(2‐propylheptyl) phthalate
DPrP: di‐n‐propyl phthalate
DQOs: daily quality objectives
DtDP: ditridecyl phthalate
DuDP: diundecyl phthalate
EPA: environmental protection agency
FSES: Food Safety and Environmental Stewardship
HMW: high molecular weight
IS: internal standard
LMW: low molecular weight
PAHs: polycyclic aromatic hydrocarbons
PCBs: polychlorinated biphenyls
PCPs: personal care products
PFAS: per‐and polyfluoroalkyl substances
PSA: primary secondary amine
SWBs: silicone wristbands
TOTM: tris(2‐ethylhexyl) trimellitate

## 1 Introduction

In response to both widespread occurrence and toxicological concerns, phthalates have become a significant focus of environmental monitoring efforts in the last decade. Phthalates have been used for the past 60 years in a wide array of consumer and industrial products [1], and are the most prevalent plasticizers in the world [2]. Phthalates may migrate into surrounding environments such as air [3], soils [4], sediments [5], and sewage [6], as well as fresh and marine/ocean waters [7, 8, 9]. In addition to environmental matrices, phthalates are commonly found in commercial goods and products, where phthalates can be bound to a plastic matrix by hydrogen bonding or Van der Waals forces which in turn allows them to leach into foods containing oils or high fat content [10, 11, 12, 13]. Concurrent with trepidation over widespread exposure, there are concerns about potential adverse health effects with phthalates [14], including hormone disruption [15], reproductive toxicity [16, 17], and developmental issues [15, 16]. In response to this research, increasing regulatory restrictions have prompted the development of phthalate alternatives or replacements, which require ever wider ranges of analytical methods for evaluation.

While all phthalates are esters of 1,2‐benzenedicarboxylic acid, they collectively exhibit diverse physicochemical properties due to variations in alkyl side‐chain lengths [18]. Phthalates are generally classified as low molecular weight (LMW) (with < six carbon chains) or high molecular weight (HMW) (with > six carbon chains) [19], with LMW phthalates primarily found in personal care products and HMW phthalates used predominantly as plasticizers [9]. In response to regulatory and health concerns, several alternatives have emerged, including isophthalates and terephthalates—structural isomers with substitutions in the meta or para positions, respectively—as well as adipates and trimellitates, which offer similar functional properties with lower toxicological risks [2, 20, 21, 22, 23, 24]. Adipates provide good product performance expectations at low temperatures due to their low viscosities [20], while tris(2‐ethylhexyl) trimellitate (TOTM) is commonly used for high temperature settings such as PVC cables [20]. Replacements for phthalates have been used for over a decade [20] and are forecast to have an increase in production from 2021 to 2025 due to the expanding regulations over phthalate health concerns [2]. Physiochemically similar phthalates and/or alternatives can be analytically challenging since their structures may lead to coelutions and require robust separation to resolve and be quantified. The class as a whole is challenging due to the abundant variety/types of phthalates in numerous products, their prevalence in background and quality control samples, and the wide range of concentrations that may be present in the sample media [10, 14, 25, 26, 27] In order to capture LMW and HMW phthalates, dynamic separation techniques such as large oven profiles could be needed. This analytical separation and resolution challenge could explain why many studies measuring personal exposures or foods typically only monitor for 11 phthalates or less and few if any replacements [26, 28, 29, 30].

Aside from analytical separation considerations, there are detection ranges and cleanup strategies that may warrant investigation. For instance, phthalates are often seen in concentrations several orders of magnitude higher than other analyte classes in environmental sampling such as organo–phosphate flame retardants, polycyclic aromatic hydrocarbons (PAHs), pesticides, polychlorinated biphenyls (PCBs), and per‐and polyfluoroalkyl substances (PFASs) [31, 32, 33, 34]. Another aspect to consider are cleanup strategies of environmental or personal samplers. One of the most common techniques to cleanup a sample prior to analysis is SPE, but the sorbent used may be especially important to consider given the similar inherent structure of phthalates [35].

The objective was to create a comprehensive phthalate analytical method using gas chromatography mass spectrometry (GC‐MS) that broadened the typical chemistries considered in previous studies while still providing adequate separation in order to achieve robust quantitation. Compound selection for this method started with 14 phthalates from a previous semi‐quantitative screening method [36, 37], and more were added from the literature including those found in indoor air [26], food contact materials [38], and outdoor contaminated sites [39]. Phthalates such as bis(2‐propylheptyl) phthalate (DPrHtP), diundecyl phthalate (DuDP), didecyl phthalate (DDP), and ditridecyl phthalate (DtDP) were included which are not often studied but could be important personal exposure compounds since they are used in a variety of applications as low and high temperature resistant items such as cables, car interior furniture, roofing membranes [40, 41, 42] and electronics [43]. In addition to compound diversity and chromatographic optimization, goals of this work also included strategies for compound ranges and quantitation, logistical considerations of sample preparation including SPE, as well as compound storage information. Some of these goals are highlighted through the extraction and use of the above method on challenging real‐world samples such as cooking oils (representing oral routes of exposure) and passive samplers worn against the skin (silicone wristbands, or SWBs, representing dermal/contact/atmospheric routes of exposure) [44, 45] Collectively, this work establishes a valuable foundation to monitor phthalates and their emerging alternatives, contributing to a broader understanding of their distribution and potential impacts.

## 2 Methods

### 2.1 Chemical Standard and Reagents

Thirty‐one standards were purchased from AccuStandard Inc. (New Haven, CT), Santa Cruz Biotechnology (Dallas, TX), Sigma‐Aldrich (Saint Louis, MO), and Toronto Research Chemical (Toronto, Canada). Table 1 contains the full list of target and laboratory surrogate analytes along with chemical abstract service (CAS) numbers and physicochemical properties. The target list for this method is a broad collection of common and less commonly studied phthalates and alternatives, composed of molecular weights ranging from 194–531 g/mol (Table 1). Three deuterated compounds, diamyl phthalate (DAP‐d4), di‐n‐butyl phthalate (DnBP‐d4), and di‐n‐nonyl phthalate (DnNP‐d4) served as internal standards (IS) and surrogates for instrument quantitation spiked at 5000 ng/mL (Toronto Research Chemical). The two surrogates (DnBP‐d4 and DnNP‐d4) are quantified from DAP‐d4 as an IS to account for any loss of recovery during extraction processes. Target analytes were then subsequently quantified from surrogate recoveries. All solvents were optima grade from Fisher Scientific (Hampton, NH), and all water used for processing was ultrapure (18 MΩ·cm) (MilliQ Advantage A10, Millipore, Saint Lewis, MO)

**TABLE 1:** Target analytes and surrogates with physicochemical properties and analytical data.

	Type	Compounds	CAS	Abbreviation	CPSIA List	Mol Wt. (g/mol)	Boiling point (°C)	Melting point (°C)	Log *K*_oa_	Log *K*_ow_	RT	SIM ion	Qualifier ions
1	**Target compounds**	Dimethyl phthalate	131‐11‐3	**DMP**		194	285	36	5.7	1.6	12.109	163	133, 194
2		Diethyl phthalate	84‐66‐2	**DEP**		222	300	−21	6.7	2.4	12.800	149	177, 105
3		Diisopropyl phthalate	605‐45‐8	**DiPrP**		250	305	5.3	7.4	2.8	13.060	149	122, 167
4		Diallyl phthalate	131‐17‐9	**DALP**		246	313	14	7.4	3.2	13.400	149	132, 189
5		di‐n‐propyl phthalate	131‐16‐8	**DPrP**		250	317	−0.17	7.4	3.3	13.523	149	191, 209
6		di‐iso‐butyl phthalate	84‐69‐5	**DiBP**	**Restricted**	278	296	22	8.2	4.1	13.840	149	104, 223
7		di‐n‐butyl phthalate	84‐74‐2	**DnBP**	**Restricted**	278	340	−34	8.8	4.6	14.150	149	205, 160
8		bis(2‐methoxyethyl) phthalate	117‐82‐8	**DMEP**		282	340	−45	8.0	1.3	14.300	149	176, 167
9		Diisopentyl phthalate	605‐50‐5	**DiPP**		306	318	−21	8.7	4.6	14.500	149	237, 104
10		bis(2‐ethoxyethyl) phthalate	605‐54‐9	**DEEP**		310	345	34	9.2	2.5	14.600	149	176, 193
11		Diamyl/dipentyl phthalate	131‐18‐0	**DAP**	**Restricted**	306	342	−21	9.4	5.6	14.750	149	306, 219
12		bis(4‐methylpentyl) phthalate	146‐50‐9	**DMPP**		334	377	−19	—	—	15.080	149	251, 167
13		di‐n‐hexyl phthalate	84‐75‐3	**DnHxP**	**Restricted**	334	329	−58	10	6.8	15.355	149	150, 233
14		Benzyl butyl phthalate	85‐68‐7	**BBP**	**Restricted**	312	370	77	10	4.8	15.448	149	206, 150
15		di(2‐ethylhexyl) adipate*	103‐23‐1	**DEHA**		371	417	−67	11	6.9	15.515	129	147, 130
16		bis(2‐butoxyethyl) phthalate	117‐83‐9	**DBEP**		366	366	8.3	10	3.6	15.940	149	193, 176
17		bis(2‐ethylhexyl) phthalate	117‐81‐7	**DEHP**	**Restricted**	391	384	−53	12	7.5	16.215	279	149, 167
18		Diheptyl phthalate	3648‐21‐3	**DHtP**		363	384	−17	11	6.5	16.215	247	149, 265
19		Dicyclohexyl phthalate	84‐61‐7	**DCHP**	**Restricted**	330	331	66	11	5.8	16.248	249	149, 167
20		Diphenyl phthalate	84‐62‐8	**DPhP**		318	399	73	11	3.6	16.390	225	226, 153
21		bis(2‐ethylhexyl) isophthalate	137‐89‐3	**DEHiP**		391	384	−27	12	7.5	17.050	149	167, 261
22		di‐n‐octyl phthalate	117‐84‐0	**DnOP**		391	397	−6.0	12	8.1	17.400	149	261, 279
23		bis(2‐ethylhexyl) terephthalate*	6422‐86‐2	**DEHT**		318	384	−48	12	7.6	17.485	225	141, 104
24		Diphenyl isophthalate	744‐45‐6	**DPhiP**		391	399	80	11	3.6	17.486	149	112, 261
25		Dibenzyl phthalate	523‐31‐9	**DBzP**		346	413	44	12	3.7	17.710	107	149, 91
26		bis(2‐propylheptyl) phthalate	53306‐54‐0	**DPrHtP**		447	417	−16	12	8.9	17.940	149	167, 307
27		di‐n‐nonyl phthalate	84‐76‐4	**DnNP**		419	429	1.5	12	7.7	18.550	149	150, 293
28		Didecyl phthalate	84‐77‐5	**DDP**		447	416	−0.9	12	9.0	19.536	149	167, 307
29		Diundecyl phthalate	3648‐20‐2	**DuDP**		475	413	−4.5	12	9.0	19.640–20.517	149	167, 321
30		tris(2‐ethylhexyl) trimellitate*	3319‐31‐1	**TOTM**		547	414	29	12	8.0	21.012	305	193, 323
31		ditridecyl phthalate	119‐06‐2	**DtDP**		531	442	−7.5	12	10	22.923	149	150, 349
**1**	**Surrogate (SS) and internal (IS) standards**	di‐n‐butyl phthalate‐d4 (SS)	93952‐11‐5	**DnBP‐d4**		282	—	—	—	—	14.150	153	209, 164
**2**		Diamyl phthalate‐d4 (IS)	358730‐89‐9	**DAP‐d4**		310	—	—	—	—	14.750	153	241, 223
**3**		di‐n‐nonyl phthalate‐d4 (SS)	1202865‐43‐7	**DnNP‐d4**		423	—	—	—	—	18.550	153	207, 297

*Note*: Compounds highlighted by an asterisk indicate replacement phthalates. A total of seven compounds in this method listed have restrictions under the consumer product safety investigation act (CPSIA) of the US. Target compounds are listed in order of retention time. Physicochemical properties gathered from EPA CompTox Database and EpiWeb 4.1.

### 2.2 Sample Extraction

Extraction methods for all samples were based on published methodology, and sample types were chosen by previously reported data on phthalates and challenges posed by the matrices. Samples of olive, vegetable, and coconut oils were taken straight from the bottle (plastic or glass) and extracted based on previous work [46]. Briefly, 1.0 g of oil was weighed in a glass vial and 10 mL of ACN was added. Next, surrogates were added at 5000 ng/mL and the mixture was vortexed for at least 15 s before centrifugation at 2500 revolutions per minute (RPM) for approximately 10 min (Eppendorf Centrifuge 5810 R, Hamburg, Germany). The resulting supernatant was pipetted into a Turbovap evaporator container and blown down to 1 mL at 40°C (Biotage LLC, Charlotte, NC). The concentrated extract cooled to room temperature and transferred to chromatography vials. IS was added at 5000 ng/mL and the extract was stored frozen (−20°C) until analysis.

SWBs are personal passive samplers shown to detect phthalates in previous work [31, 32, 33], and were gathered from past internal studies that were known or likely to contain phthalates but not analyzed on this current method [29, 47, 48, 49]. Briefly, wristbands were prepared prior to deployment by conditioning at 280°C under vacuum (∼ 0.1 torr) as previously described [32, 45, 50, 51]. Samplers were stored at −20°C after deployment, and each SWB was solvent extracted with two rounds of ethyl acetate (∼100 mL) with surrogates added at 5000 ng/mL prior to extraction to account for analyte loss during extraction process [45]. Decanted solvents were combined and blown down to 1 mL using Biotage evaporators. Ultimately sample SPE cleanup was utilized using primary secondary amine (PSA) cartridges (see Supporting Information for SWB SPE details). All cleaned extracts were then stored at −20°C until analysis after adding IS at 5000 ng/mL.

### 2.3 GC‐MS Optimization

This method was developed on an Agilent 8890 5977 GC‐MS in select ion monitoring (SIM) mode, equipped with a 3 mm extractor lens and an Agilent J&W 30 mm × 0.250 mm × 0.25 µm DB‐5MS column (part number 122–5532, Agilent Technologies, Santa Clara, CA). Injection was done using an Agilent 7396A Automatic liquid sampler. A 1 µL was injected into an Agilent Ultra Inert 4‐mm single taper GC liner, manually packed with deactivated glass wool, with an injection temperature of 290°C in pulsed splitless mode. Glass wool often can reduce potential quantitation differences between standards used in calibration and real‐world samples [52], but care was taken to consistently packed the deactivated glass wool to avoid unintentional variance in the analysis [52]. The thermal auxiliary (MSD transfer line) heater was set at 300°C and the source and quadrupole temperature were at 300°C and 180°C respectively. Helium carrier gas was set at a constant flow rate of 1.157 mL/min.

As each compound was added to the method, dwell times were adjusted to achieve Gaussian peak shape with a minimum of 15 cycles across all peaks. Target compounds were identified by converging lines of evidence including relative retention time, signal to noise ratio that is at least (≥ 3:1), peak shape, comparison to a reference mass spectrum, and at least one confirmation ion being within the expected response ratio. As compounds were identified, specific SIM windows were created for collections of targets throughout the run to increase resolution and sensitivity. The oven program for this method was optimized for separation by having a starting temperature of 40°C for 2 min, ramping 10°C/min to 100°C, ramping at 25°C/min to 200°C, ramping 40°C/min to 280°C with a 3‐min hold, and finally ramping 20°C/min to 335°C with a 7‐min hold (see Table S1 in Supporting Information initial and final instrument conditions). A 5‐min post run follows at 340°C. The total run time was optimized to achieve adequate separation (Table S1) and was finalized at 31.75 min. All GC‐MS data was analyzed with Agilent Mass Hunter software (version 10.0, Wilmington, DE).

### 2.4 Method Performance: Calibration

Each target compound was quantified from at least a 6‐point calibration curve with a coefficient of determination (*r* ^2^) > 0.98. With the diversity of analytes in this method, it was decided to use one of two calibration curves depending upon sensitivities and concentrations reported in the literature. For instance, median concentrations observed in environmental exposure matrices ranged from 100 to 2510 ng/g in food [46, 53], 470 to 1 809 000 ng/g in dust [3, 54], and 11 000 to 4 070 000 ng/g in some personal care products [55] among phthalates in this method. Similarly, in previous SWB samples from this laboratory, concentrations for eight phthalates (BBP, DnBP, DEP, DiBP, DnHxP, DCHP, DnOP, and DEHA) ranged from 225 to 45 000 ng/g [31, 32, 33]. Based on these data, each compound was assigned to a concentration range (i.e. 250–10 000 ng/mL or 2000–25 000 ng/mL, see Table S2 for compound assignment).

### 2.5 Method Performance: LODs and Inter‐Day Variability

Repeated analyses of calibration standards were used to calculate both LODs and inter and intra‐day variabilities for each analyte using calibrant standards. Reporting limits were based on EPA methods where the LODs are calculated with at least seven replicates [40], and in this work sensitivity studies were performed by running two concentration standards (250 and 2000 ng/mL) a total of 12 times across three consecutive days. The lowest appropriate calibration level was used for each analyte (see Table S2 for calibration assignment). For LODs, the standard deviation was then multiplied by the *t*‐value (99% confidence level) to establish limits for each analyte [41]. LOQs were created by multiplying the LOD value by five for compounds that were quantitated from either curve. The 250 ng/mL standard was also used to determine inter‐ and intra‐day variability for all targets and was analyzed 15 times over 3 days.

### 2.6 Solid Phase Extraction

Sample cleanup with SPE is often necessary for complex matrices but can be challenging with this compound class with background contamination or processing. In this study, all SWB samples were processed with SPE cleanup to reduce residual skin oils that could interfere with analysis similar to other work [49]. To begin method development, C18 SPE cartridges were purchased from Agela Technologies (Torrance, CA) and florisil and PSA were purchased from Agilent Technologies (Wilmington DE). Solvent spike replicates (*n* = 4) were spiked at 15 000 ng/mL to evaluate phthalate recovery for all three cartridge types in addition to comparisons of blank cartridges to examine background phthalates inherent with the cartridges.

### 2.7 Phthalate Compound Storage

As part of method validation objectives, solvent standards (*n* = 4) were evaluated for recovery at 0, 12, 50, and 133 days. Standards were stored under refrigeration (∼4°C) in between injections. Sample caps were replaced after injection to reduce evaporation between evaluations.

### 2.8 Quality Control

On the instrument, a continuing calibration verification solution was run before and after of every batch of samples (ex: 10–15 samples) to verify that the instrument was in good operating condition along with instrument blanks to confirm no carryover or instrument contamination between sample sets. Contamination of phthalates can commonly occur and can happen at any processing step within the procedure [56]. Some of these sources of contamination can be from lab tools, sampling, sample preparation, solvents, clean‐up, extraction, laboratory air, or the instrument itself [57, 58]. To avoid reporting false positive data, it is important to include blanks at every processing stage to correct for potential contamination. During sample processing, solvent extraction blanks, blank wristbands (conditioned but unworn by participants), and instrument blanks were included to capture phthalates and subsequent concentrations for background correction for both oils and SWBs. Any background found was accounted for in the data presented below. For an example of this background in blank SWB, see Figure S1. In addition, blank SWBs were also evaluated to see if silicone interfered with phthalate quantitation by overspiking the subsample extract just before instrument injection at 1000 ng/mL. Figure S2 has percent recoveries of three blank SWB matrix spikes which ranged from 17% to 145%, but with a median of 80% (Figure S2). Only three phthalates were outside 30% of expected values (DDP, DnNP, and TOTM) and are noted in Table 2 below.

**TABLE 2:** Average detected phthalate and alternative concentrations (ng/g) in edible oils and silicone wristbands (SWBs) from this and other studies.

Edible oils	All values in ng/g	Number of targets	DEP	DALP	DPrP	DBP	DMEP	DEHA	DBEP	DEHP	DBzP	DuDP
**Olive oil**												
	**Olive oil plastic (*n* = 3)**	**31**	156	BDL	BDL	BDL	BDL	361	1560	805	BDL	BDL
	**Olive oil glass (*n* = 3)**	**31**	BDL	494	BDL	BDL	2680	BDL	BDL	834	2180	BDL
	**Cavaliere et al. [** 60 **] (*n* = 8)**	**7**				210				940		
	**Arena et al. [** 61 **] (*n* = 20)**	**9**			815					324		
	**Ferracane et al. [** 62 **] (*n* = 23)**	**7**	52		411	557				271		
**Vegetable oil**												
	**Vegetable oil (*n* = 3)**	**31**	BDL	BDL	BDL	BDL	BDL	262	2870	< 200	1130	152
	**Arena et al. [** 61 **] (*n* = 7)**	**9**								230		
	**Liu et al. [** 63 **] (*n* = 5)**	**7**				280				510		
**Coconut oil**												
	**Coconut oil (*n* = 3)**	**31**	BDL	BDL	BDL	BDL	BDL	922	BDL	< 200	286	BDL
	**Kiralan et al. [** 46 **] (*n* = 2)**	**6**				100				750		

Silicone WBs	All Values in ng/g	Number of targets	DMP	DEP	DiBP	DnBP	DnHxP	BBP	DEHA	DEHP*	DHtP*	DEHiP	DnOP	DEHT*	DPrHtP	DnNP^	DDP^	DuDP*	TOTM*^	DtDP
**Children**																				
	**Urban children (*n* = 3)**	**31**	830	6080	1310	46.7	327	4710	195 000	36 000	3100	60 000	BDL	240 000	BDL	12 100	570	6000	1290	10 000
	**Rural children (*n* = 3)**	**31**	870	3610	3000	10 800	BDL	4530	32 000	103 000	BDL	BDL	BDL	266 000	BDL	26 100	BDL	2940	1880	14 200
	**Hammel et al. [** 28 **] (*n* = 77)**	**19**	22.6	739	976	275		1060	1000	9010				8490						
**Adults**																				
	**Urban adult (*n* = 5)**	**31**	BDL	3670	1020	42.0	BDL	1660	8880	74 900	BDL	BDL	904	48 100	BDL	BDL	BDL	2630	542	8210
	**Young et al. (USA) (*n* = 85)**	**6**			1000	500				5000				23 000						
	**Young et al. (UK) (*n* = 42)**	**6**			500	500				4000				16 000						
	Young.(China)(*n* = 70)	**6**			5000	1000				28 000				13 000						
	**Young** **et al. (India) (*n* = 54)**	**6**		3000	1500	2000				36 000				18 000						
**Occupational**																				
	**Roofers (lapels) (*n* = 4)**	**31**	20.0	56.7	BDL	BDL	BDL	33.3	113	1190	BDL	BDL	BDL	210	BDL	66.7	BDL	16.7	197	403
	**Roofers (WB) (*n* = 3)**	**31**	643	1520	957	BDL	127	287	2260	9220	440	BDL	BDL	1510	153	1060	110	2560	1860	3750
	**Craig et al. [** 29 **] (lapels) (*n* = 9)**	**13**							5.00	251				81.9						
	**Craig et al. [** 29 **] (WB) (*n* = 9)**	**13**							2.30	42.4				20.8						

*Note*: Rows with other comparison studies have results converted to ng/g if not already presented in those units and averaged for comparative purposes. *Compounds DEHP, TOTM, DHTP, DuDP, and DEHT may be impacted with storage stability accuracy as mentioned in the text, while ^denotes compounds that may have been altered by silicone matrix.

*Abbreviation*: BDL, below detection limit. Young et al. ‐ A. A. S. Young, N. Herkert, H. M. Stapleton, et al., “Chemical contaminant exposures assessed using silicone wristbands among occupants in office buildings in the USA, UK, China, and India,” Environment international 156 (2021): 106727.

## 3 Results and Discussion

### 3.1 GC‐MS Optimization

**FIGURE 1:**
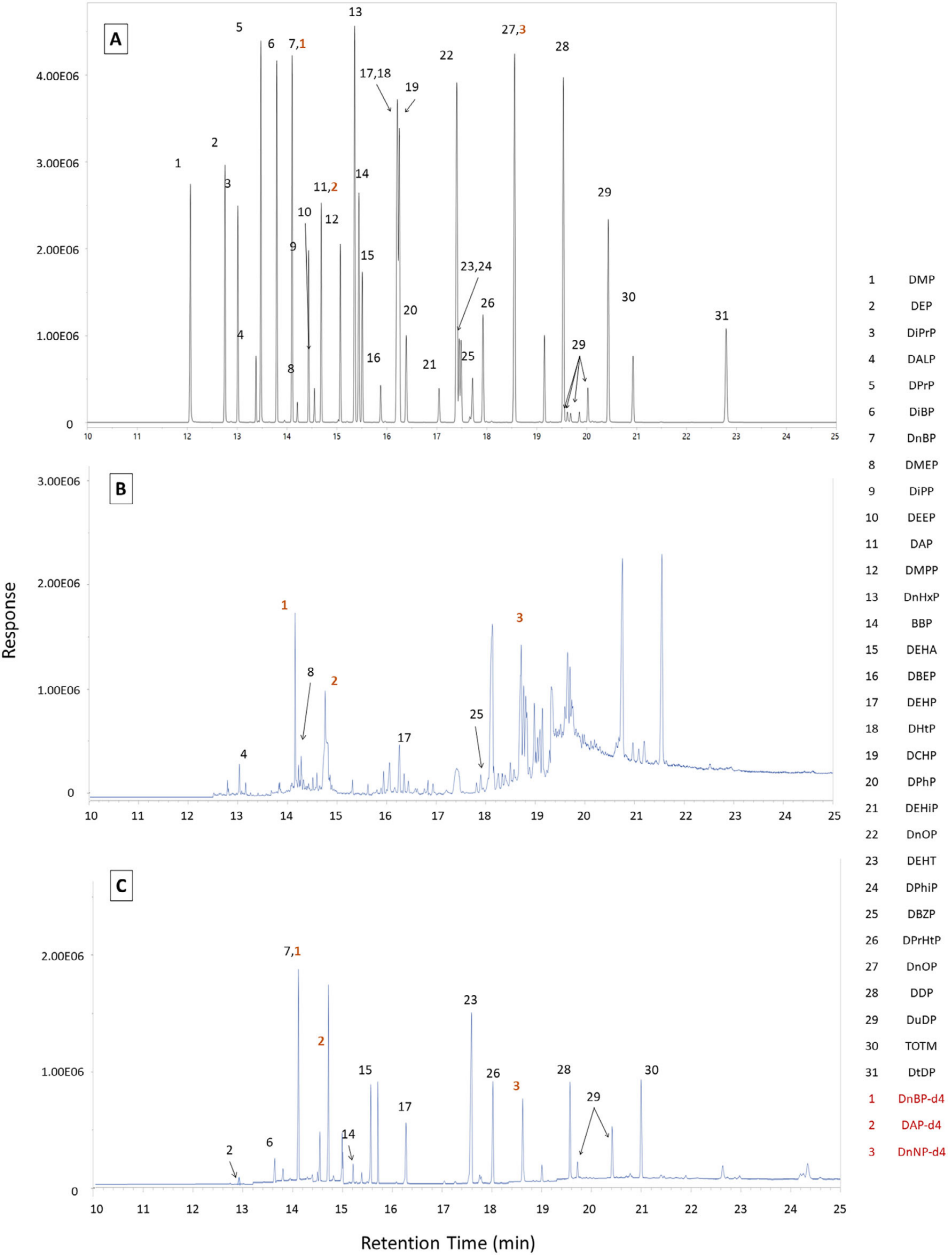
SIM GC‐MS chromatographs showing: (A) solvent standard of a mixed concentration of 25 ppm and 10 ppm that displays resolution of 27 target phthalates, three replacements, and three deuterated compounds (red numbers), (B) a representative olive oil sample from a glass container; (C) a representative SWB sample worn by a child in an urban environment. Coelution peaks (which were still mass‐resolved) are marked with all compounds in the peak.

Despite many investigations in the literature for phthalates, only a small subset of investigations contains 15 or more phthalates per analysis. Of those, this study has one of the broadest ranges of phthalates (excluding replacements) in terms of boiling points (271°C–517°C) and octanol partitioning (log*K*_ow_: 1.5–10) as well as several unique phthalates (see Table S3). When developing the method to incorporate both phthalates and replacements based on an earlier publication [26], initial chromatography displayed several challenges: two sets of coelutions; fronting on the LMW compounds; and considerable peak broadening for the HMW compounds such as DtDP and TOTM. The first set of coelutions were between DEHP, DHtP, and DCHP, and the second set between DEHiP and DEHT. The first series of modifications to the oven profile (holds, ramp rates, additional ramps, etc.) collectively improved peak shape overall, particularly for the HMW phthalates (see Table S4 for chronological modifications). In addition, chromatographic resolution between coelutions was also improved from the modifications resulting in an average of 25**%** increase of resolution between peaks (see Figure 1A—SIM scan chromatogram for all peaks). In addition, all compounds that displayed a coelution were able to be fully mass‐resolved. This was the result of selecting unique SIM fragment ions so each compound could be quantified separately (see Table 1 for quantifying ions). In order to quantitate some of the HMW phthalates with optimal peak shape, the maximum oven profile temperature was set at 335°C. As real‐world SWB samples were analyzed by the method, less SWB matrix carry over by 25% was noticed with an additional 5‐min hold at 340°C post‐run.

### 3.2 Compound Ranges and Quantitation

#### 3.2.1 Edible Oils—Modest Concentration Ranges

**FIGURE 2:**
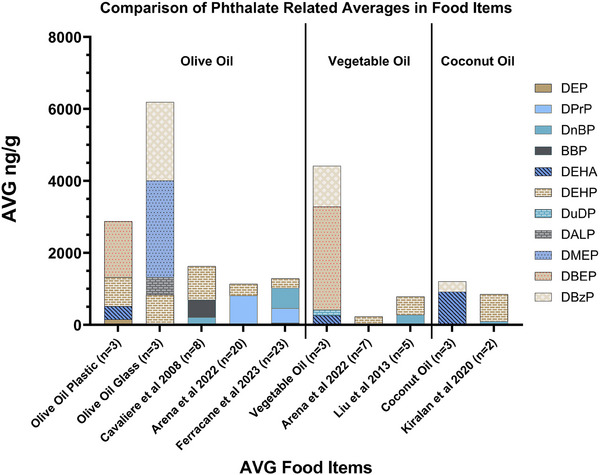
Method comparison to known phthalate‐containing oils. Stacked bar graphs of average detected phthalate concentrations in ng/g found in edible oils from this study compared with values from the literature. Although the number of phthalates analyzed in each graph and study may be different, this comparison shows values of phthalates in the same magnitude from those exacted in this study to those from the literature. This comparison was to demonstrate the capabilities and comparison of this method to other published methods and was not expected to recreate exact concentrations considering different source material, methodology, and phthalates analyzed.

The concentration ranges of phthalates observed in our analyses (hundreds to thousands of nanograms per gram (ng/g)) are consistent with those reported in the literature, though differences in compound selection across studies limit direct comparison. All edible oils tested contained detectable levels of phthalates (see Figure 1B for a representative SIM chromatogram), with the most commonly detected phthalate being DEHP (Figure 2). In Figure 2, phthalate profiles can be compared from this study to profiles other oils of the same type. All oils had multiple phthalates detected, with DEHP, DEHA, and DBzP detected in all or most types of samples (see Table 2 for all average detections). These phthalates were also detected in 20 cooking oils [46] from the literature and are commonly reported in food related products [59] (see Table S5 for more phthalates sources). The ranges of detected concentrations were 150–2870 ng/g, again similar to ranges of concentrations reported in coconut oil (100–700 ng/g) [46], olive oil (180–800 ng/g) [60, 61, 62], and vegetable oil (200–500 ng/g) [61, 63] (see also Table 2). Interestingly, two less commonly detected phthalates (DMEP and DBEP) were seen in olive oil from a glass container and plastic cap (DMEP—2680 ng/g), and in olive oil from a plastic container (DBEP—1560 ng/g, Figure 2). These phthalates are less commonly used in application and production (Table S5 for individual phthalate sources), but are present here in concentrations similar to or higher than DEHP indicating the importance of studying a wider range of phthalates [64].

#### 3.2.2 Solid‐Phase Extraction of Silicone Wristbands and Phthalates

Although post‐extraction SPE cleanup was not used for all samples in this study, all of the SWB samples were processed with SPE to remove fatty acids, oils and other potential interferences in the instrumental analysis [32]. However, due to the similar complexity of lipids and phthalates, SPE processes that specialize in fatty acid removal can also interfere with phthalate recoveries [57]. Several common cleanup phases were tested including C18 cartridges based on prior studies [32, 45], florisil [28, 65], and PSA [66]. The goal was a cleanup method that would help reduce maintenance on the instrument but also keep recoveries of the most phthalates within acceptable criteria for real‐world samples (± 30%). While C18 proved effective for the LMW phthalates, C18 was ineffective for the HMW compounds (Figure S3). Specifically, HMW phthalates DDP, DuDP (all associated peaks), TOTM, and DtDP were completely removed (i.e., <LOD) after C18 SPE (Figure S3). This loss is likely due to Van der Waals interactions between the C18 phase and the HMW compounds in the sample. In addition, C18 SPE blank samples had background levels for 14 phthalates (Table S6). In contrast, florisil and PSA cartridges had average percent recoveries of at least 90% (vs. 70% average with C18) including adequate recovery of HMW phthalates (Figure S3). PSA had an average percent recovery of 96% while florisil averaged 90% across all compounds. In addition, PSA had slightly lower RSD than florisil (2.5% and 5.3%, respectively). Both cartridge types had lower background levels of phthalates detected than C18 cartridges (Table S6). Based on method objectives for reducing biological matrices with high fatty acid content, and/or silicone content from the SWBs, the PSA SPE cartridge was chosen for the SWB sample set having the most phthalates meet our DQOs (28 of 31) (Figure S3).

#### 3.2.3 Personal Sampling Silicone Wristbands—Large Concentration Ranges

**FIGURE 3:**
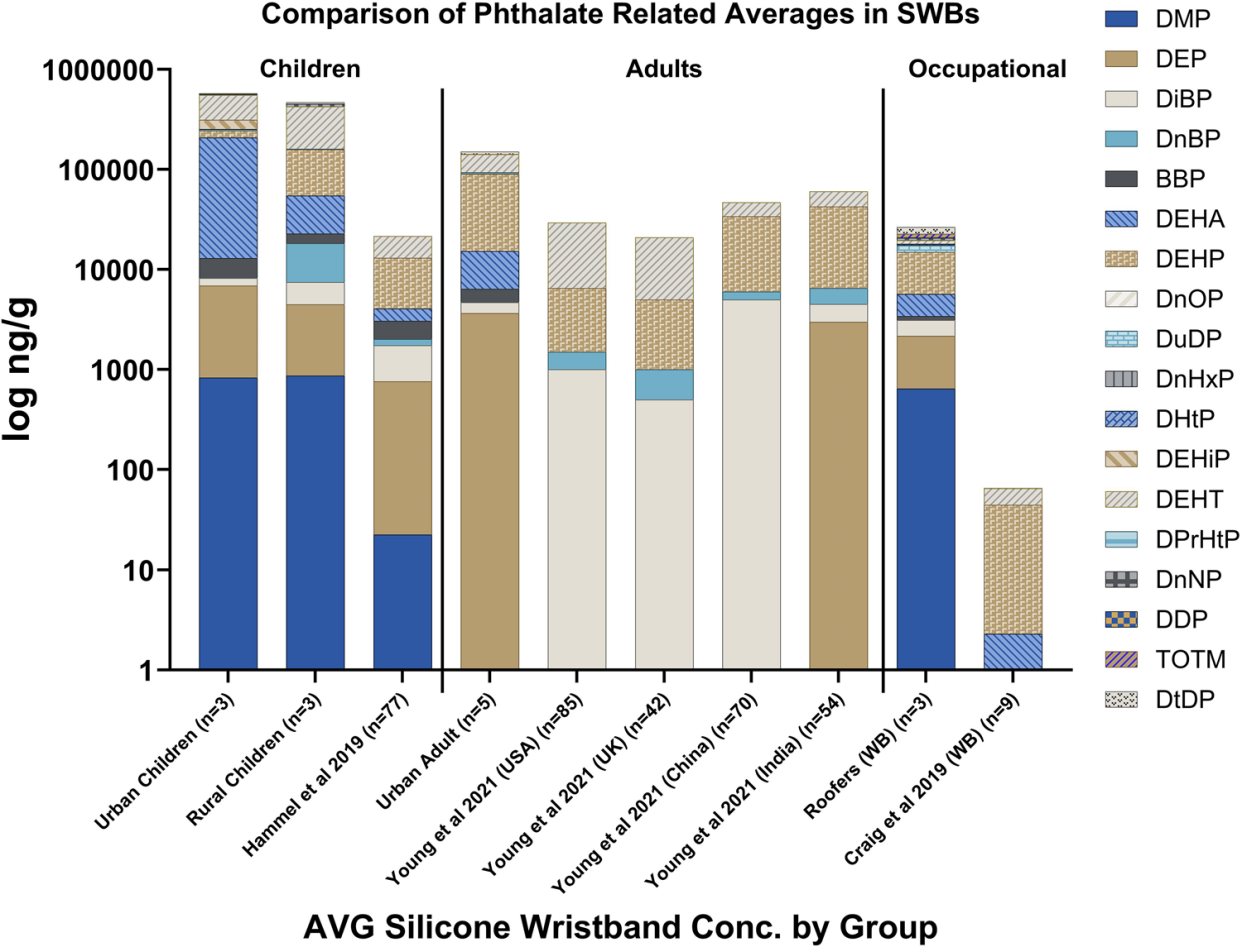
Profile comparisons of phthalates from human exposures. Stacked bar graphs of average detected concentrations of individual phthalates (ng/g) found in SWBs worn by groups of people from internal laboratory studies compared with a values found in SWBs from the literature. Note that the *y*‐axis is in log10 scale in order to see the profiles of pthalates between groups of individual averages. Cautious comparisons between phthalates of different bars should be made because of the logarithmic scale. Young et al. 2021 ‐ A. S. Young, N. Herkert, H. M. Stapleton, et al., "Chemical contaminant exposures assessed using silicone wristbands among occupants in office buildings in the USA, UK, China, and India," Environment international 156 (2021): 106727.

Ranges of phthalates seen among the SWB samples spanned nearly five orders of magnitude from 16 ng/g (DuDP) to over 265 000 ng/g (DEHT). This again stresses the importance of calibration ranges. The highest levels were seen from DEHA, DEHT, and DEHP, often with values over 100 000 ng/g. Other studies analyzing phthalates in SWBs have also reported high concentrations of DEHP and DEHT, often in similar magnitudes (Figure 3). In order to quantitate many of these samples that have high phthalate content, serial dilutions were necessary, particularly for DEHA, DEHP, and DEHT. This was true for wristbands worn by preschool age children (DEHT: 132 000–306 000 ng/g and DEHA: 16 400–498 000 ng/g), rural youth farmworkers (DEHT: 164 000–380 000 ng/g, and DEHP: 54 900–145 000 ng/g), and pregnant individuals (DEHA: 5500–14 800 ng/g, DEHT: 11 800–104 000 ng/g, and DEHP: 20 600–235 000 ng/g). In contrast, having a lower level of sensitivity was important for common phthalates like DMP, DnHxP, DnOP, and DDP which were detected in average concentrations of 20–900 ng/g, as well as less commonly detected phthalates such as DPrHtP and DuDP which were seen at levels between 60–6000 ng/g (Table 2). Even a single sample can be challenging for the range of concentrations detected. For instance, a preschool age child's SWB had a range of 40–240 000 ng/g within a single sample. This finding warrants more study for a value that high for a relatively understudied phthalate.

Aside from concentration ranges, SWB samples showed at least 10 unique phthalates detected in each group (see Figure 3, and see Figure 1C for a representative SIM chromatogram). Two phthalates were seen in all SWB samples, DEHP and DEHT (Figure 3). Several others were detected in all internal groups of SWBs including DEP, DiBP, BBP, DEHA, DuDP, TOTM, and DtDP (Table 2). When comparing profiles of stacked average concentrations between this study and others, similar patterns emerge especially for children having detectable levels of DMP and DEP in all study groups (Figure 3). Both of these are found in personal care products among other common sources (see Table S5 for more source information).

### 3.3 Storage Stability

While most phthalates were stable over time, some compound responses do require careful attention when storing standards for more than a month under refrigeration. Recovery and variability were assessed by injecting a set of three aliquots of a mixed standard after 12, 50, and 133 days, (Figure S4). In total, 23 out of 31 compounds were within data quality objectives (± 30% recovery and < 15% RSD) after all timepoints, with some phthalate signals becoming enhanced or reduced over time, particularly at later time points (Figure S4). After 50 days, most compounds still met DQOs, but recoveries ranged from 63.1%–233% with an average of 94.7%, and a median of 83.2%. DEHP had the highest percent recovery at 50 days (233%), but several other compounds had over 150% recoveries as well (DHtP, DCHP, and TOTM). RSDs were still robust and ranged from 0.5%–16% with an average of 2.0% with DuDP with the highest RSD (16%) and lowest recovery (63.1%). DuDP breaks down into five peaks, so it was expected that it might have the lowest recoveries and higher variability relative to the rest of the analytes. At the 133‐day mark, similar trends were seen as the 50‐day mark with most recoveries (23 out of 31) within 30% (Figure S4) and averaged RSDs below 10%. DEHT had the lowest recovery (31%) and highest variability (30%), while DEHP, DHtP, DCHP, and TOTM all again having inflated recoveries over 130%. Inflated recoveries could derive from phthalates leaching from cap liners or other contamination known to occur, especially for DEHP [58]. Special attention to these five phthalates and replacements (DEHP, DEHT, DHtP, DCHP, and TOTM) should be considered when working with these standards in future studies to ensure accuracy and precision.

## 4 Conclusions

This method is a targeted analysis for a large number of phthalates that can be used in conjunction with challenging matrices that require extra processing or cleanup. To the authors’ knowledge, this method quantifies at least four phthalates not commonly looked for in other studies and contains one of the biggest ranges of chemical diversity among the phthalates quantified. Together, this methodology and accompanying considerations for extraction provides a detailed framework and foundation for future phthalate methodology.

As manufacturing patterns change for plasticizers, it will be increasingly important to expand phthalate analyses and especially phthalate replacement analyses in consumer goods or in personal monitoring. In 2020, phthalates were responsible for 55% of world consumption of plasticizers down from 60%–65% a few years ago [2]. The decrease in phthalate consumption is mainly due to regulations in countries including the USA, China, and the European Union reducing or banning phthalates in cosmetics, toys, or food packaging, [2, 16, 27, 67]. These recent restrictions has led to an increase in phthalate replacement consumption such as trimellitates and some aliphatics [2], with limited toxicological data on replacement plasticizers [20]. Future work should continue to expand along with trends in commerce.

## Author Contributions

**Kaley T. Adams**: investigation, methodology, validation, writing – original draft, writing – review and editing. **Caoilinn Haggerty**: investigation, methodology, resources, validation, writing – original draft, writing – review and editing. **Richard P. Scott**: investigation, methodology, validation, writing – review and editing. **Steven O'Connell**: conceptualization, project administration, visualization, writing – original draft, writing – review and editing. **Kim A. Anderson**: conceptualization, funding acquisition, supervision, validation, writing – review and editing. All authors read and approved the final manuscript.

## Conflicts of Interest

K. A. A. and S. O., two authors of this research, disclose a financial interest in MyExposome, Inc., which is marketing products related to the research being reported. The terms of this arrangement have been reviewed and approved by Oregon State University in accordance with its policy on research conflicts of interest. The authors have no other disclosures.

## Supporting information




**Supporting information file 1**: jssc70227‐sup‐0001‐SuppMat.docx

## Data Availability

Many sample measurements are available in this published article and its Supporting Information files. Any other datasets analyzed during the current study are available from the corresponding author on reasonable request.
