# Supplementary material for: Targeted Gas Chromatography‐Mass Spectrometry Analysis of 31 Phthalates and Replacements: Method Optimization and Application to Edible Oils and Silicone Wristbands
Source: J Sep Sci. 2025 Jul 17;48(7):e70227. doi: 10.1002/jssc.70227 (PMC12272031; doi:10.1002/jssc.70227)
Supplement: Supplementary file 1 — Supporting information file 1: jssc70227‐sup‐0001‐SuppMat.docx [file JSSC-48-e70227-s001.docx]

# Supporting Information – Considerations for phthalate extraction and analysis using a comprehensive GC-MS method

### Table of Contents (in order of appearance in MS):

Silicone Wristband - SPE cleanup

Table S1: GC-MS Instrument Conditions: Initial and final oven profiles

Table S2: Calibration ranges, reporting limits and reproducibility

Figure S1: Blank wristband background

Figure S2: Blank SWB overspike recovery

Table S3: Individual phthalate comparisons among comprehensive methods

Table S4: Chronological Oven Profile Modifications

Table S5: Common Uses/Sources of all phthalates

Figure S3: Comparison of the C18, Florisil, and PSA SPE cartridges

Table S6: SPE Background for C18, PSA, and Florisil Cartridges

Figure S4: Stability Recovery after 12, 50, and 133 Days

### Silicone Wristband - SPE cleanup

Prior to phthalate analysis, extract aliquots were further cleaned with solid phase extraction (SPE) using 3 mL 250 mg Primary Secondary Amine (PSA) cartridges (Agilent Technologies, Wilmington DE). Each cartridge was conditioned with ACN prior to sample loading (> 2mL). An aliquot of 50 µL of sample was added to 1.5mL of ACN and loaded on the cartridge. The sample was then eluted with an additional 1mL of ACN using a positive pressure manifold (Thermo Fisher Scientific, Waltham, MA) with nitrogen gas and an adjusted pressure to achieve 1-2 drops per second. Samples were then re-concentrated to 50 µL and solvent exchanged to ethyl acetate.

Table S1: GC-MS Instrument Conditions: Initial and final oven profiles.

An initial ramp was added to improve chromatography. A 3 min hold was added to the third ramp to improve the peak shape of the high molecular weight compounds.Despite further improvements, the HMW compounds still displayed poor chromatographic peak shape until the addition of a of temperature ramp from 320 to 335 °C at 20 °C/min where we were able to improve the peak width at ½ height for DtDP and TOTM by 20%. A post run hold was added to reduce matrix carry over. No instrument hardware was changed.

Table S2: Calibration ranges, reporting limits and reproducibility for all compounds. The reporting limits here include what is calculated with real matrices and potential interferences. The peak number for each compound corresponds to Figure 1 in the manuscript.

Table S2 continued. Compounds highlighted in red indicate replacement phthalates. Target compounds are listed in order of retention time.


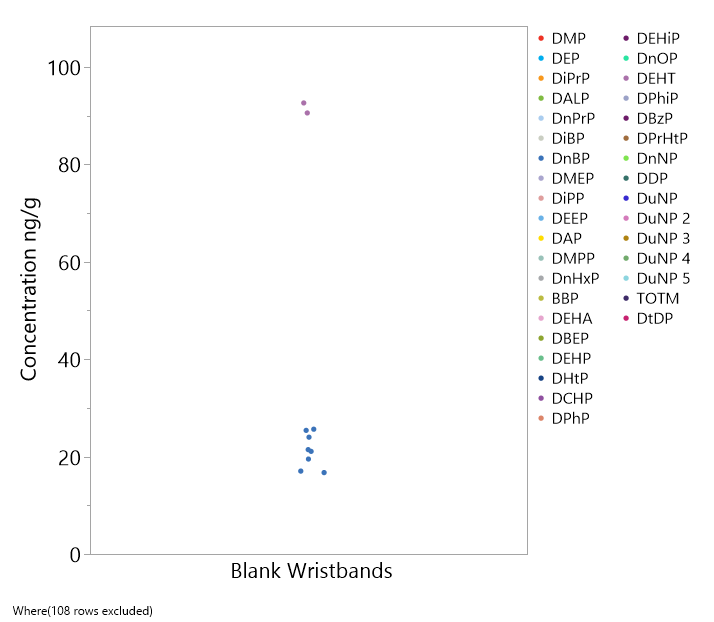


Figure S1: Blank wristband background.

Blank wristbands (n=8) from multiple lots were analyzed and only showed two compounds detected: DnBP (average concentration of 21 ng/g, blue dots) and DEHT (average concentration of 92 ng/g, purple dots). The values for both compounds were consistent with RSDs of 7.5% for DnBP and 1.1% for DEHT. These values were background subtracted from the values reported in the MS.


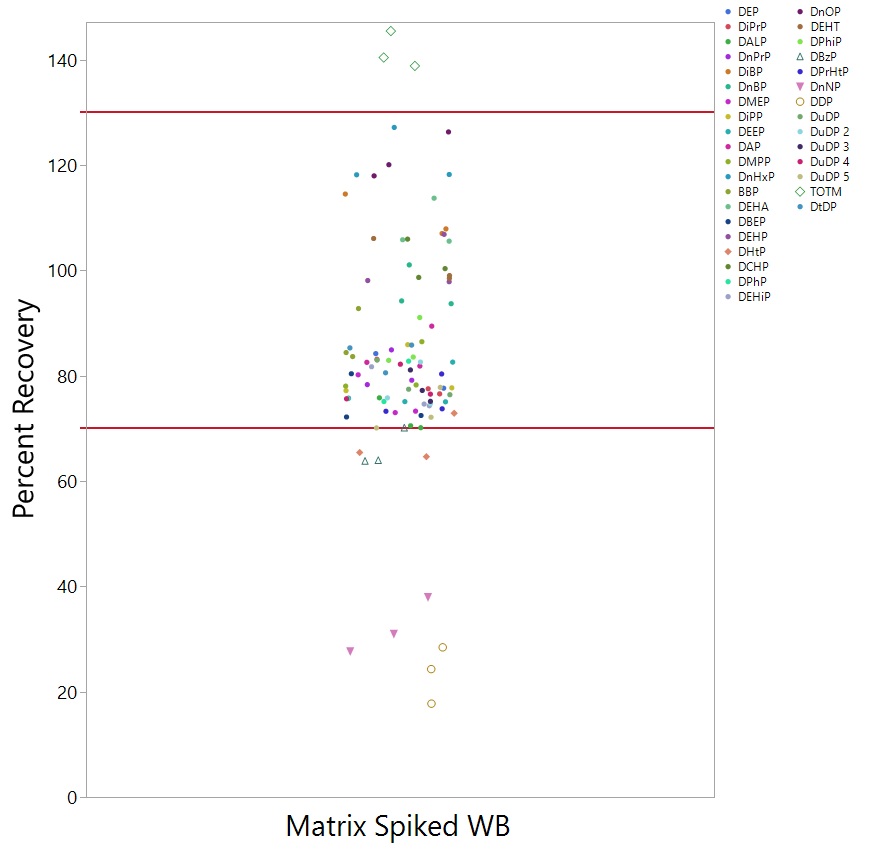


Figure S2: Matrix Spiked Recovery of SWBs.

Conditioned wristbands were spiked (n=3 at 1000 ng/ml) with all target compounds in the method. Average percent recovery was 83%, with a median of 80% and an RSD of 8.1%, with a total range of 17-145% (DDP and TOTM respectively). Red Lines are the DQO’s. Although compound groupings outside of DQOs were not mathematically adjusted in real samples, compounds with values outside of these recoveries are noted in the associated table of data in the manuscript.

Table S3: Individual phthalate comparisons among papers with at least 15 phthalates per method.

Red bold “x” indicates unique phthalates (excluding replacement phthalate compounds) from this method compared to those in the table (n=4). Physicochemical parameters were obtained from EPA Comptox (TEST and OPERA predictive models) and dashed lines indicate no data were available for that compound. Boiling points (BP) are listed in Celsius. Each study has the total number of phthalates listed underneath the citation.

Table S4: Oven Profile Modifications

Table S4 shows the step-by-step individual modifications that were made in the process of building this method from the beginning oven profile taken from Takeuchi et al 2014. The bold text shows exactly wat was modified and the results with each change are listed afterwards.

Table S5: Common Sources of all phthalates

Table S5 lists out some of the common sources for each phthalate as documented in the literature. Common sources of many phthalates include personal care products (PCP), polyvinyl chloride (PVC), building materials and food packaging. [1], [2], [3], [4], [5], [6], [7], [8], [9], [10], [11], [12], [13], [14], [15], [16], [17], [18], [19], [20].

[1] Liang, Y., Caillot, O., Zhang, J., Zhu, J., Xu, Y., Large-scale chamber investigation and simulation of phthalate emissions from vinyl flooring. *Building and Environment*. 2015, *89*, 141-149.

[2] Wittassek, M., Koch, H. M., Angerer, J., Brüning, T., Assessing exposure to phthalates–the human biomonitoring approach. *Molecular nutrition & food research*. 2011, *55*, 7-31.

[3] Craig, J. A., Ceballos, D. M., Fruh, V., Petropoulos, Z. E., Allen, J. G., Calafat, A. M., Ospina, M., Stapleton, H. M., Hammel, S., Gray, R., Exposure of nail salon workers to phthalates, di (2-ethylhexyl) terephthalate, and organophosphate esters: a pilot study. *Environmental science & technology*. 2019, *53*, 14630-14637.

[4] Young, A. S., Allen, J. G., Kim, U.-J., Seller, S., Webster, T. F., Kannan, K., Ceballos, D. M., Phthalate and organophosphate plasticizers in nail polish: evaluation of labels and ingredients. *Environmental science & technology*. 2018, *52*, 12841-12850.

[5] Houlihan, J., Brody, C., Schwan, B., Not too pretty. *Phthalates, Beauty Products and the FDA*. 2002.

[6] Hines, C. J., Nilsen Hopf, N. B., Deddens, J. A., Calafat, A. M., Silva, M. J., Grote, A. A., Sammons, D. L., Urinary phthalate metabolite concentrations among workers in selected industries: a pilot biomonitoring study. *Annals of occupational hygiene*. 2009, *53*, 1-17.

[7] Bui, T. T., Giovanoulis, G., Cousins, A. P., Magnér, J., Cousins, I. T., de Wit, C. A., Human exposure, hazard and risk of alternative plasticizers to phthalate esters. *Science of the total environment*. 2016, *541*, 451-467.

[8] Poitou, K., Rogez-Florent, T., Lecoeur, M., Danel, C., Regnault, R., Vérité, P., Monteil, C., Foulon, C., Analysis of Phthalates and Alternative Plasticizers in Gloves by Gas Chromatography–Mass Spectrometry and Liquid Chromatography–UV Detection: A Comparative Study. *Toxics*. 2021, *9*, 200.

[9] Schütze, A., Gries, W., Kolossa-Gehring, M., Apel, P., Schröter-Kermani, C., Fiddicke, U., Leng, G., Brüning, T., Koch, H., Bis-(2-propylheptyl) phthalate (DPHP) metabolites emerging in 24 h urine samples from the German Environmental Specimen Bank (1999–2012). *International journal of hygiene and environmental health*. 2015, *218*, 559-563.

[10] Hubinger, J. C., A survey of phthalate esters in consumer cosmetic products. *Journal of cosmetic science*. 2010, *61*, 457-465.

[11] Stroustrup, A., Bragg, J. B., Busgang, S. A., Andra, S. S., Curtin, P., Spear, E. A., Just, A. C., Arora, M., Gennings, C., Sources of clinically significant neonatal intensive care unit phthalate exposure. *Journal of exposure science & environmental epidemiology*. 2020, *30*, 137-148.

[12] Schettler, T., Human exposure to phthalates via consumer products. *International journal of andrology*. 2006, *29*, 134-139.

[13] Meng, X., Zhang, N., Sun, X., Niu, Z., Deng, Y., Xu, J., Bai, H., Ma, Q., Suspect screening of 200 hazardous substances in plastic toys using ultra-high-performance liquid chromatography-hybrid quadrupole time-of-flight mass spectrometry. *Journal of Chromatography A*. 2020, *1617*, 460830.

[14] Sapozhnikova, Y., Hoh, E., Suspect Screening of Chemicals in Food Packaging Plastic Film by Comprehensive Two‑Dimensional Gas Chromatography Coupled to Time‑of-Flight Mass Spectrometry. 2019.

[15] Grodzinski, J., New Method for Determining Phthalate Esters in Propellants. *Analytical Chemistry*. 1955, *27*, 1765-1767.

[16] Gries, W., Ellrich, D., Küpper, K., Ladermann, B., Leng, G., Analytical method for the sensitive determination of major di-(2-propylheptyl)-phthalate metabolites in human urine. *Journal of Chromatography B*. 2012, *908*, 128-136.

[17] Weschler, C. J., Indoor-outdoor relationships for nonpolar organic constituents or aerosol particles. *Environmental science & technology*. 1984, *18*, 648-652.

[18] Autian, J., Toxicity and health threats of phthalate esters: review of the literature. *Environmental health perspectives*. 1973, *4*, 3-26.

[19] Gooch, J. W., in: Gooch, J. W. (Ed.), Encyclopedic Dictionary of Polymers. Springer New York, New York, NY 2011, pp. 217-217.

[20] <https://pubchem.ncbi.nlm.nih.gov/compound/Ditridecyl-phthalate> (last time accessed: April 15).


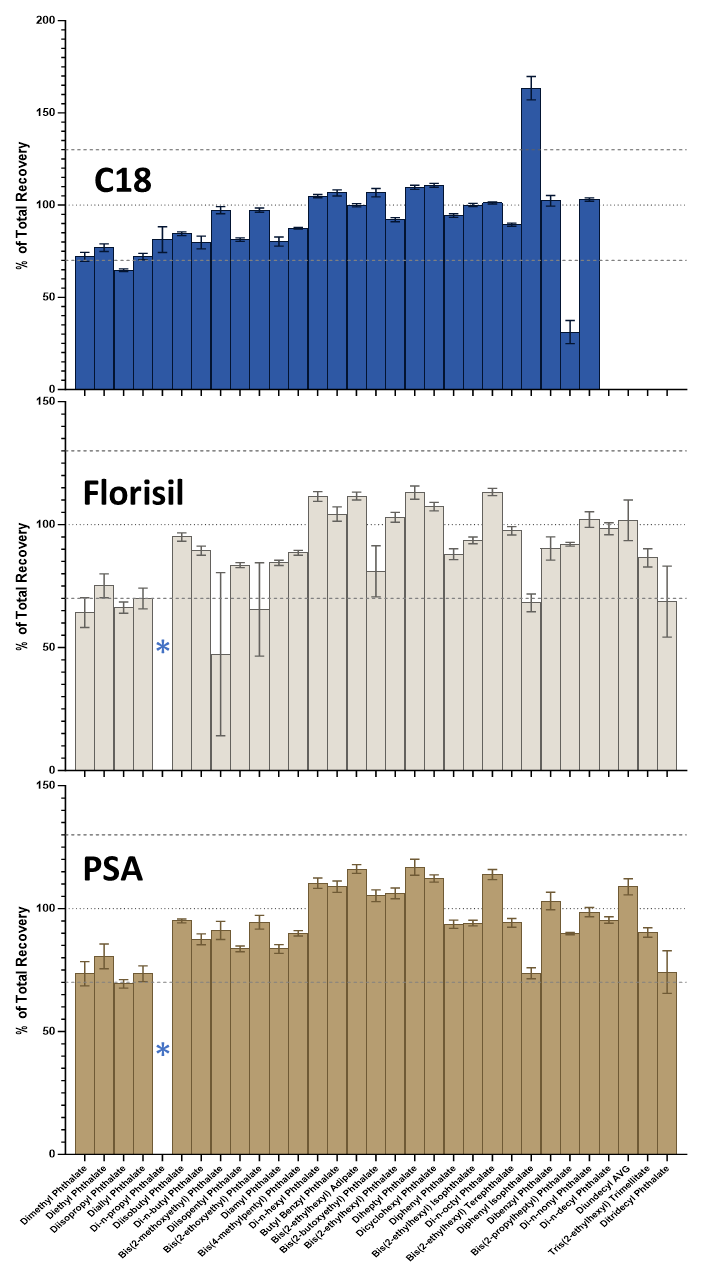
Figure S3: Comparison of the C18, Florisil, and PSA SPE cartridges for phthalate recovery (n=4) at 15,000 ng/mL. Dashed lines are located at 70 and 130% recovery, while a dotted line is located at 100% recovery for reference. Significant loss is seen using C18 cartridges (blue bars, HMW compounds from didecyl phthalate (DDP) to ditridecyl phthalate (DtDP) were not detected). Blue stars indicate that di-n-propyl phthalate was detected for both Florisil and PSA but 15,000 ng/mL is over its calibration range (0.25ppm-10ppm). Florisil had an average of 90% recovery, PSA had 96% recovery, and C18 had 70% recovery. Only 3 compounds didn’t meet our internal DQO’s with PSA (+/- 30% recovery), while 8 compounds for Florisil and 11 for C18 did not meet criteria.

Table S6: SPE Background for C18, PSA, and Florisil Cartridges

|  |  | DEP | DiPrP | DPrP | DiBP | DBP | DiPP | DHxP | BBP | DEHA | DEHP | DOP | DEHT | DBzP | DPrHP | TOTM |
| --- | --- | --- | --- | --- | --- | --- | --- | --- | --- | --- | --- | --- | --- | --- | --- | --- |
| C18  n=1 | Avg Conc. (ng/mL) | <BDL | 95.6 | 105 | 507 | 881 | 129 | 547 | 227 | 1357 | 1501 | 666 | 822 | 208 | 309 | 1047 |
| PSA  n=1 | Avg Conc. (ng/mL) | 52.7 | 99.5 | <BDL | <BDL | 235 | 125 | 506 | 109 | <BDL | 409 | 600 | 322 | <BDL | 202 | <BDL |
| Florisil  n=1 | Avg Conc. (ng/mL) | <BDL | 101 | <BDL | <BDL | 226 | 126 | <BDL | <BDL | <BDL | 403 | <BDL | <BDL | <BDL | <BDL | <BDL |

All concentrations are in ng/ml. There were a significant number of phthalates seen in SPE blanks. C18 SPE cartridges were purchased from Agela Technologies (Torrance, CA) and florisil and PSA were purchased from Agilent Technologies (Wilmington DE) and were made out of plastic. In the C18 cartridges, there were 14 detections for phthalates ranging from 96-1500 ng/mL. The PSA cartridges had 10 hits for phthalates from 52.7 to 600 ng/mL. The Florisil cartridges had only 4 hits for phthalates from 100 – 403 ng/ml. While PSA had more background than Florisil, it still met more DQOs across the phthalate list and was chosen for SPE in this study

Figure S4. Phthalate stability recovery over time under refrigeration (0, 12, 50, 133 days). Data quality objectives were +/- 30% for recovery (dashed lines) and less than 15% RSDs. Compounds DEHP, DEHT, DCHP, DEHT and TOTM had high % recoveries, with DEHP having the highest at 50 days. DEHT had the lowest % recovery at 133 days. At the 12-day mark, RSD%’s ranged from 0.6%-5.0%. RSD%’s ranged from 63%-233% after 50 days. Overall, most compounds were stable throughout the time period, but as stated in the main text, a few phthalates (DEHP, DHtP, DCHP, DEHT, and TOTM) should be monitored for external contamination or loss over time as appropriate.
